# Role of the Arabidopsis PIN6 Auxin Transporter in Auxin Homeostasis and Auxin-Mediated Development

**DOI:** 10.1371/journal.pone.0070069

**Published:** 2013-07-29

**Authors:** Christopher I. Cazzonelli, Marleen Vanstraelen, Sibu Simon, Kuide Yin, Ashley Carron-Arthur, Nazia Nisar, Gauri Tarle, Abby J. Cuttriss, Iain R. Searle, Eva Benkova, Ulrike Mathesius, Josette Masle, Jiří Friml, Barry J. Pogson

**Affiliations:** 1 Australian Research Council Centre of Excellence in Plant Energy Biology, College of Medicine, Biology and Environment, Research School of Biology, The Australian National University, Canberra, Australia; 2 Research School of Biology, The Australian National University, Canberra, Australia; 3 School of Life Science and Biotechnology; Heilongjiang August First Land Reclamation University, Daqing, China; 4 Department of Plant Systems Biology, VIB and Department of Plant Biotechnology and Genetics, Ghent University, Gent, Belgium; 5 Institute of Science and Technology Austria (IST Austria), Klosterneuburg, Austria; 6 School of Molecular and Biomedical Sciences, School of Agriculture, Food and Wine, The University of Adelaide, Adelaide, Australia; Wake Forest University, United States of America

## Abstract

Plant-specific PIN-formed (PIN) efflux transporters for the plant hormone auxin are required for tissue-specific directional auxin transport and cellular auxin homeostasis. The Arabidopsis PIN protein family has been shown to play important roles in developmental processes such as embryogenesis, organogenesis, vascular tissue differentiation, root meristem patterning and tropic growth. Here we analyzed roles of the less characterised Arabidopsis PIN6 auxin transporter. PIN6 is auxin-inducible and is expressed during multiple auxin–regulated developmental processes. Loss of *pin6* function interfered with primary root growth and lateral root development. Misexpression of *PIN6* affected auxin transport and interfered with auxin homeostasis in other growth processes such as shoot apical dominance, lateral root primordia development, adventitious root formation, root hair outgrowth and root waving. These changes in auxin-regulated growth correlated with a reduction in total auxin transport as well as with an altered activity of DR5-GUS auxin response reporter. Overall, the data indicate that *PIN6* regulates auxin homeostasis during plant development.

## Introduction

The plant hormone auxin is involved in embryogenesis, organogenesis, vascular tissue differentiation, and hypocotyl and root elongation, as well as growth responses to environmental stimuli. Auxin is synthesized in the shoot, leaf tips as well as in the root apex, and transported to other parts of the plant. Auxin signaling requires the interplay of auxin biosynthesis, conjugation, transport and perception, and can be modified through complex interactions with other hormones (see reviews by [1], [2], [3]). Auxin moves in a polar manner from cell to cell or through the phloem from source to sink tissues. The basipetal auxin flow occurs from the shoot apex to the base and contributes to maintaining apical dominance. In roots, auxin moves both acropetally through the central stele and basipetally through the epidermis and outer cortex near the root tip [4], [5]. Polar cell-to-cell auxin transport is mediated by carrier proteins of the AUX1 (Auxin resistant 1), LAX (like-AUX1), PGP (Phospho-glycoprotein) and PIN (pin-formed) families [3]. While AUX1, LAX and certain PGP-type proteins are involved in auxin import [6], [7], [8], there are other PGP-type and PIN proteins that mediate auxin export from the cell [6], [9].

The Arabidopsis genome encodes eight PIN proteins and their sequences differ mainly in the central hydrophilic region. PIN proteins 1, 2, 3, 4 and 7 contain a large hydrophilic loop, PIN6 contains a partially reduced loop, while PIN5 and PIN8 lack the central hydrophilic domain [9], [10], [11]. Accordingly, phylogenetic analysis in Arabidopsis indicated that the PIN family can be divided into two subclasses, the plasma membrane (PM) localized PIN1-type (PIN1, 2, 3, 4, 7) and the endoplasmic reticulum (ER) localized PIN5-type (PIN5, 6, 8) [10], [12] that appear functionally distinct. The asymmetric localization of the plasma membrane-localized PIN proteins determines the direction of inter-cellular auxin flow [13].

PIN proteins function to coordinate vascular tissue differentiation and regeneration [14], [15], meristem maintenance and patterning in roots [16], [17], [18] as well as organ development in general [19], [20]. The loss-of-function *pin1* mutant shows the most extensive phenotype as it grows a pin-shaped floral stem inflorescence devoid of floral organs due to lower auxin transport [14], [21]. Mutations in *pin2* (or ethylene insensitive root 1; *eir1*) reduced root waving, and the *PGP1* and *PGP19* phosphoglycoprotein (PGP) auxin transporters [22], [23] as well as the *WAG1/WAG2* PINOID related protein-serine/threonine kinases, which are critical factors in PIN membrane polarity establishment, also affect root waving [24], [25], [26], [27]. However, whether there are particular roles for specific PIN proteins in root waving is not well documented or understood.

The phenotypes associated with the loss-of-function of PIN1, and to lesser extent PIN2, 3, 4 and 7 can be phenocopied by some synthetic auxin transport inhibitors [9], [28], [29], [30]. The best characterized auxin-associated phenotypes are dose-dependent effects on shoot apical dominance and branching, the emergence of root hairs, primary root elongation, lateral root initiation and emergence as well as responses to gravity [31], [32], [33], [34], [35]. The *PIN* genes have functions linked to organogenesis (PIN1), pattern formation during embryogenesis (PIN1, PIN4 and PIN7) and phototropic responses as well as various forms of tropism (PIN2 and PIN3) [16], [28], [30], [36], [37]. The ER localised PIN5 and PIN8 proteins mediate auxin exchange between the ER lumen and cytosol and play an important role in the control of the ER-resident auxin metabolism [10], [38], [39]. PIN6 is the last member of this developmentally important gene family and despite being recently shown to have a function in the development of Arabidopsis stamens and nectary production, its function in mediating auxin growth processes remains functionally uncharacterised [40].

Here we show that PIN6 is a crucial component of auxin transport and auxin homeostasis and contributes to auxin-dependent growth and development processes such as root and shoot growth as well as reproductive development.

## Materials and Methods

### Plant Growth Conditions and Transgene Selection

Seeds were cold-stratified in the dark for 3 days and grown at 21°C under a 16 hour photoperiod (150 µmol quanta m^2^ s^1^ irradiance), unless otherwise stated. Sterilised seeds were grown on Murashige and Skoog (MS) media (4.4 g/L MS salts and vitamins, 1% sucrose, 0.8% agar). Transgenic Arabidopsis (Col-O) lines harbouring *pMDC32::PIN6* or *TPIN6::FiLUC* were selected on MS media containing 50 µg/mL hygromycin (Invitrogen) or 50 mg/L BASTA (Sigma-Aldrich), respectively.

### Mutants Used in this Study

All germplasm were in the *Arabidopsis thaliana* ecotype Columbia (Col-0) background. Germplasm used in this study include TDNA insertion lines [Salk_046393 (*pin6-2*), Salk_092831 (*pin6-4*), GABI-KAT_430B01 (*pin6-5*) and GK711C09 (*pin6-6*)].

### Construction of PIN6 Overexpression, Promoter-reporter Gene as Well as RNA Silencing Constructs and Selection of Representative Transgenic Lines

A PIN6 overexpression vector was created by PCR amplifying a *PIN6* (AT1G77110) genomic fragment (−4bp 5′UTR, 3554 bp gene and+119 bp 3′UTR) using high fidelity Platinum *Pfx* Taq Polymerase and primers (Table S1) according to the manufacturer’s instructions (Invitrogen). The *PIN6* genomic product was cloned into the pMDC32 overexpression vector [41] using Gateway® Technology to create pMDC-35s::*PIN6*. A PIN6 promoter sequence (−705 bp) thought to contain most of the upstream regulatory domain was amplified using DYNAzyme Polymerase and primers (Table S1), according to instructions (FINNZYMES). The promoter fragment was cloned into ZERO Blunt® TOPO® (INVITROGEN) intermediate vector creating TOPO::PIN6. The PIN6 promoter was subsequently cloned into pTm35::FiLUC (digested with *Xba*I/*Nco*I to remove the minimal *CaMV 35S* promoter) creating pTPIN6::FiLUC. A promoter-GUS reporter fusion was created by amplifying a much larger upstream region (−3763 bp; primers listed in Table S1) of the PIN6 promoter fragment and cloning the amplicon into pMDC162 [41] using Gateway® Technology (Invitrogen) to create pMDC-PIN6::GUS. pPIN6::GUS, pTm35enh::FiLUC and pTm35::FiLUC were prepared as previously described [20], [42].

Binary constructs were transferred into Agrobacterium strain LBA4404 and transformed into *Arabidopsis thaliana* (Col-0) using conventional techniques [43]. Selection criteria for choosing representative transgenic lines were based upon; 1) a consensus in the promoter’s tissue specific reporter gene expression pattern, 2) strength of reporter gene activity levels in leaf tissues, and 3) a consistent 3∶1 Mendelian segregation of selectable marker genes. Representative lines are indicated for pTPIN6::FiLUC (Fig. S1A), pMDC-PIN6::GUS (Fig. S2B, 2C) and pPIN6::GUS [20].

### Root Waving Phenotyping

Surface sterilised seeds were sown onto the surface of agar plates (1.5% agar supplemented with modified Hoagland solution, 1% sucrose, 0.5% MES; pH = 5.7) and cold stratified for 3 days. The plates were then placed vertical in a controlled growth chamber with setting defined above. The position of the root tip was marked daily to derive root elongation rates and 10 DAG plates were scanned enabling measurements of root length, wave length (distance between 2 successive crests) and wave amplitude (orthogonal distance between wave crest and root axis) using the ImageJ software. These measurements were used to calculate root spatial and temporal waving frequencies. Root length measurements correspond to developed lengths (determined by following the paths of each root, including along waves) of at least 12 seedlings per line in three successive experiments.

### Auxin Transport Assays

Pulse chase auxin transport experiments were performed as described [44]. Four to twelve independent basal inflorescence stem sections, mean length 30 mm, were cut from 30-day-old plants. Inverted sections were pulsed for 60 min by immersion in 20 µl 400 nM ^3^H-IAA (1 mCi.ml-1; Amersham Biosciences, UK), rinsed with 400 nM unlabelled IAA and chased with 20 µl un-labelled 400 nM IAA (150 min). Stem sections were cut into 3 mm long sections which were extracted into Ecolite scintillant overnight and radioactivity in each segment was analyzed by scintillation counting.

### Auxin Root Growth Assays

One component of auxin homeostasis is the conjugation of IAA to different moieties, including linkage to amino acids like IAA-Alanine (IAA-Ala). This type of auxin is compartmentalized in the ER and therefore a sensitivity test to IAA-Ala can reveal the extent to which auxin homeostasis has been perturbed [45]. Seedlings were transferred 3 days after germination to MS agar containing media supplemented with IAA (10, 50 and 100 nM) or IAA-Alanine (10, 20, 30µM) and relative elongation rate was determined as percentage of the control treatment (EtOH). Experiments were performed twice with similar results.

### Plant Phenotyping

Non-destructive measurements of plant growth were performed using a plant image capture and analysis system (Scanalyzer 3D, LemnaTec, Würselen, Germany; http://www.lemnatec.de/ scanalyzer_gh.htm). For root apical meristem (RAM) size measurements, 7 DAG roots were stained with propidium iodide and visualised by confocal microscopy. Root meristem size was defined as the distance from the quiescent centre to the first cells displaying elongation. For stage distribution of lateral root development, 15–20 roots from 9-day-old seedlings were processed. Lateral root primordia density was analyzed as previously described [46]. Experiments were performed twice, with a similar phenotypic outcome.

### Chlorophyll Assays

Chlorophyll measurements were performed as previously described with the following modifications: pigments were extracted in 80% acetone, and absorbance was measured at 647, 664 and 750nm [47], [48], [49].

### Real Time Quantitative PCR

Total RNA extraction, first strand cDNA synthesis and quantification of relative transcript levels were performed as previously described [50]. *Cyclophilin* (At2g29960) was used to normalise for biological variation in cDNA loading and primer sequences are listed in Table S1. Three technical replicates were performed and the results shown are representative of two independent experiments.

### β-glucuronidase and Luciferase Reporter Gene Assays

β-Glucuronidase and luciferase assays were performed as previously described [51], [52], [53].

## Results

### PIN6 Exhibits Tissue and Organ Specific Expression Patterns

The tissue specific regulation of the *PIN6* promoter (−3763 bp, −1794 bp or −705 bp) was investigated in detail using β-glucuronidase and firefly luciferase as reporters of gene expression. A large number of *PIN6* promoter-reporter transgenic lines were generated (Figs. S1 and S2), and detailed analyses are shown for several lines. In young seedlings, *PIN6* reporter expression was weak in the primary root, hypocotyl, shoot apex as well as cotyledons (Fig. S1B). Basal levels of *PIN6* expression were also observed in specific tissue types observed from mature plants, when compared to the constitutive CaMV 35S promoter (Fig. S1C). The *PIN6*::reporter lines enabled 20-fold lower leaf reporter activities when compared to the strong CaMV 35S-reporter lines (Fig. S1A and S2B). These experimental findings were consistent with anatomical gene expression analysis performed using Genevestigator, which confirms that *PIN6* is lowly expressed in most seedling tissues (Fig. S2A). Furthermore, *in silico* analysis of published microarray data showed that during development mRNA expression levels of the ER-targeted subclade of PIN transporters (*PIN5* and *PIN8*) as well as *PIN6*, are generally less abundant than those of the PM targeted PIN transporters *PIN1, 2, 7, 3* and *4* (Fig. S2D). Interestingly, the chromatin modification mark of histone H3 lysine 27 trimethylation (H3K27) that is associated with low levels of gene expression [54], was enriched surrounding the promoter and/or gene coding regions of the most weakly expressed *PIN* genes (*PIN8, 5, 6, 1* and *2*) (Fig. S2E).

**Figure 1:**
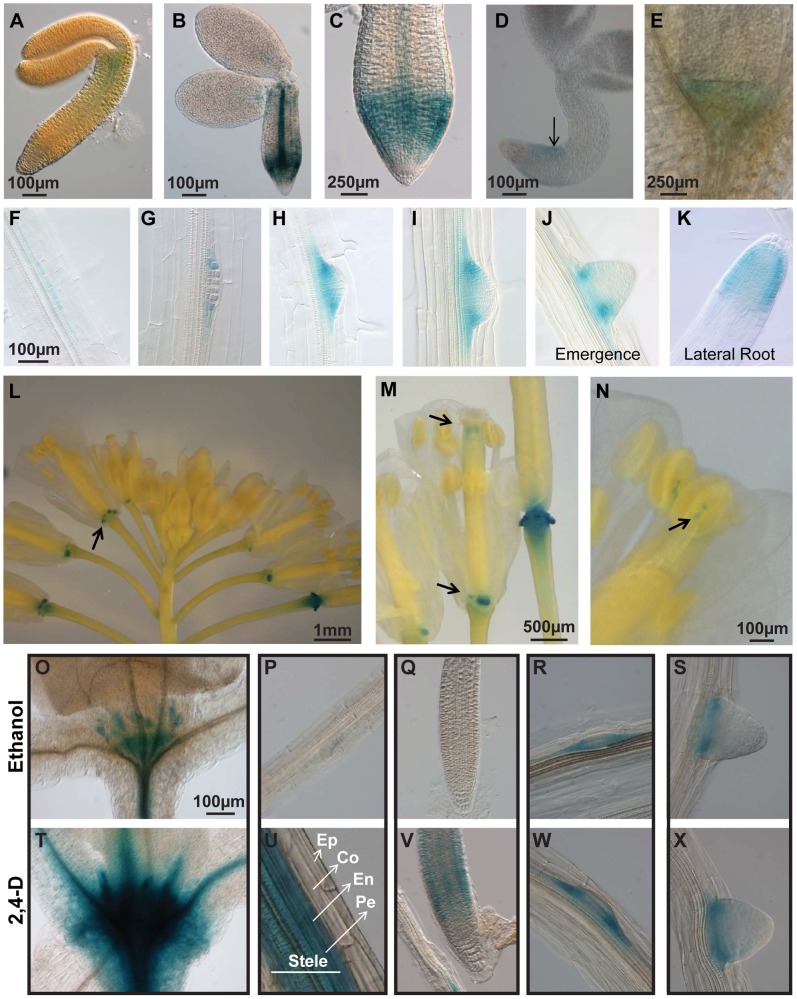
Regulation of *PIN6* expression during plant development. Representative transgenic reporter lines harbouring −1794 bp (pPIN6::GUS; A–N) of the *PIN6* promoter fused to GUS. GUS activity was observed early during germination in fresh seeds (A), dried seeds (B), the root apical meristem of seedlings (C; 1DAG), regions of asymmetric root growth (D; 2 DAG) and in the SAM (E; 3 DAG). The *PIN6* promoter enabled GUS expression during lateral root initiation (F), at the boundaries of developing root primordia (G to J) and in the lateral RAM (K), floral organ boundaries (L, M) and anthers (N). Scale bars for G–K are similar to F. O) to X) Auxin up regulates *PIN6* promoter activity. Scale bars for P-S and U–X are similar to O. Auxin treatment (2 hrs) of pPIN6::GUS transgenic lines with 10 µM 2,4-D (T–X) induces GUS expression in the SAM (T) and root (U and V), but not in the lateral root primordia (W and X). Controls (O–S) were treated with ethanol only. Epidermis (Ep), cortex (Co), endodermis (En) and Pericycle (Pe).

The expression of *PIN6* is not only developmentally regulated, but also specific of particular tissues in a cell-type specific manner (Fig. 1 and Fig. S2). During germination weak *PIN6*::GUS staining was observed in the embryos from fresh seeds and became considerably stronger in dried seed embryos (Fig. 1A, B). GUS activity was observed near the shoot meristem, root tip (RAM) and root vasculature (Fig. 1B to D). In general, there was a noticeable decline in *PIN6* reporter expression during seedling development. Later during seedling development, PIN6 promoter activity was very low in the main root meristem and vasculature (Fig. 1P and Q). *PIN6,* was however, expressed during lateral root initiation, becoming restricted to the margins of the developing primordium later during lateral root development (Fig. 1F to J) as shown previously [20]. After lateral root emergence, *PIN6* promoter activity was strongest in the outer cell layers of the lateral root meristem (Fig. 1K). Most, interestingly, the floral stem tissues showed higher reporter activity levels (Fig. S2B with S2C), which is consistent with an obvious spike in *PIN6* mRNA levels observed in floral bolts during a *PIN* developmental expression profile (Fig. S2D) [55]. Finally, the *PIN6* promoter enabled reporter expression in flowers (Fig. S1D), in particularly the nectaries and the floral organ boundaries of the anthers (Fig. 1L to N).

We next investigated the effect of auxin on *PIN6* promoter activity. Auxin stimulation (10 µM 2,4-D) induced PIN6::GUS staining in the shoot apical meristem (Fig. 1T), the root stele (including pericycle and endodermis) near the hypocotyl (Fig. 1U), and RAM (Fig. 1V). In lateral root primordia the induction of PIN6::GUS activity by auxin treatment was less obvious (Fig. 1W and 1X). This is in line with the presence of auxin response elements in the *PIN6* promoter, including AS1-like (TGACG; −91 bp, −839 bp), AuxRE (TGTCTC; −1292 bp, −993 bp) and NDE (CATATG; 1262 bp) [56], [57], [58]. In summary, the *PIN6* promoter activity is observed specifically in different developmental processes and can be induced by auxin.

### Loss-of Pin6 Function Affects Root Growth

**Figure 2:**
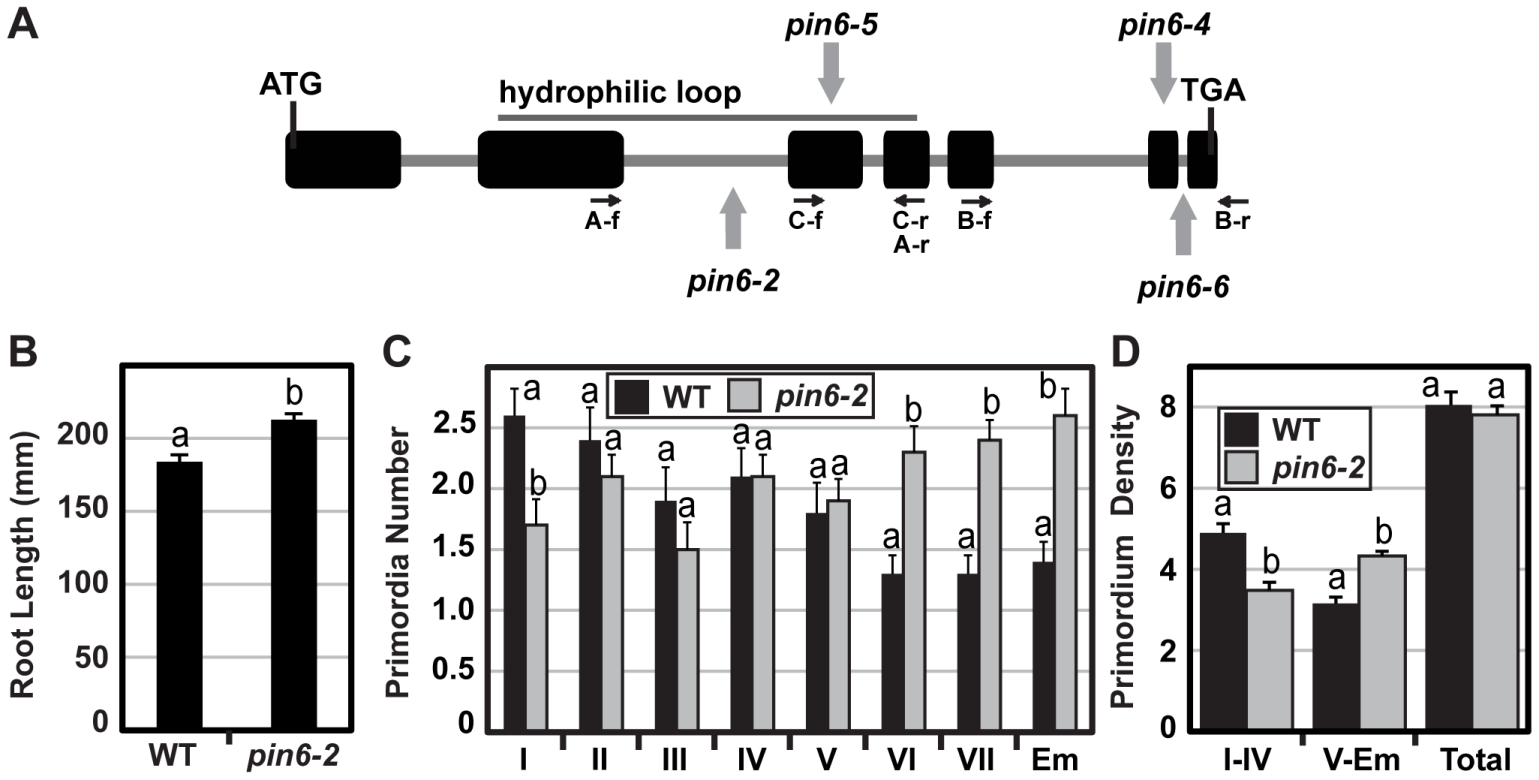
Characterisation of *pin6* mutant alleles. A) Schematic diagram of the PIN6 genomic DNA fragment showing exon/intron structure (ATG and TGA refer to translation start and stop codons, respectively). PIN6 T-DNA insertions and primer positions (See Table S1 for sequences) are marked by grey arrows. The Salk_046393 (*pin6-2*), Salk_092831 (*pin6-4*), GABI-KAT_430B01 (*pin6-5*) and GK711C09 (*pin6-6*) T-DNA insertions interrupt intron 2, exon 6, exon 3 and intron 6, respectively. B) Primary root length at 9 DAG (n = 25). (C) The development stage distributions of lateral root primordia that have initiated (I–VII) or emerged (Em) were quantified at 9 DAG (n = 10). D) The density (number of primordia per cm of primary root) of lateral root primordia in early (I–IV) and later (V–Em) stages of development, as well as the total number of primordia (n = 10). Similar results were observed in separate experiments. Homogeneity groups according to Student’s *t* test (p<0.05) are denoted with same letters. Error bars represent standard error.

We identified homozygous *pin6* mutants for three T-DNA insertion alleles (SALK_092831, GABI-KAT_430B01 and GK711C09 referred herein as *pin6-4*, *pin6-5* and *pin6-6,* respectively) (Fig. 2A). *PIN6* mRNA levels were not reduced in *pin6-4*, but partially reduced in *pin6-5* suggesting a weak loss-of-function allele (Fig. S3A). *pin6-6* represents a stronger loss-of-function mutant as *PIN6* transcripts were not observed flanking the intronic-T-DNA insertion (Fig. S3B), although *PIN6* transcript levels were still observed in a region upstream from the TDNA insertion using primers that span the third intron (Fig. S3C and D). A portion of the PIN6 protein including the hydrophilic loop may still be translated in *pin6-6*, however the removal of at least 22 amino acids form the 3′ end of the PIN6 protein could perhaps only partially disrupt protein function.

The hypocotyl and primary root lengths of *pin6* mutant alleles were similar to wild type with a slight reduction of root length in the *pin6-6* allele (Data not shown). Compared to WT, the weaker *pin6-4* and *pin6-5* mutant alleles as well as as well as the stronger *pin6-6* allele all showed a similar number of primordia at all seven stages of lateral root primordia development and primordium density was similar to wild type (Data not shown).

The recently reported *pin6-2* allele represents a complete loss-of-function allele of *PIN6* [40]. Root growth was significantly enhanced in this line (Fig. 2B). When the number of lateral root primordia was calculated, we found that there was no difference when compared to WT. However, a shift in the developmental stages of the different lateral root primordia was observed with less primordia in the early stages and more primordia and emerged lateral roots in the later stages (Fig. 2C and D), indicating a faster lateral root development.

In conclusion, the data reveal that the complete loss of *pin6* function interferes with auxin regulated growth processes in the root, in particularly lateral root development.

### PIN6 Overexpression Results in Stunted Growth and Reduces Apical Dominance

**Figure 3:**
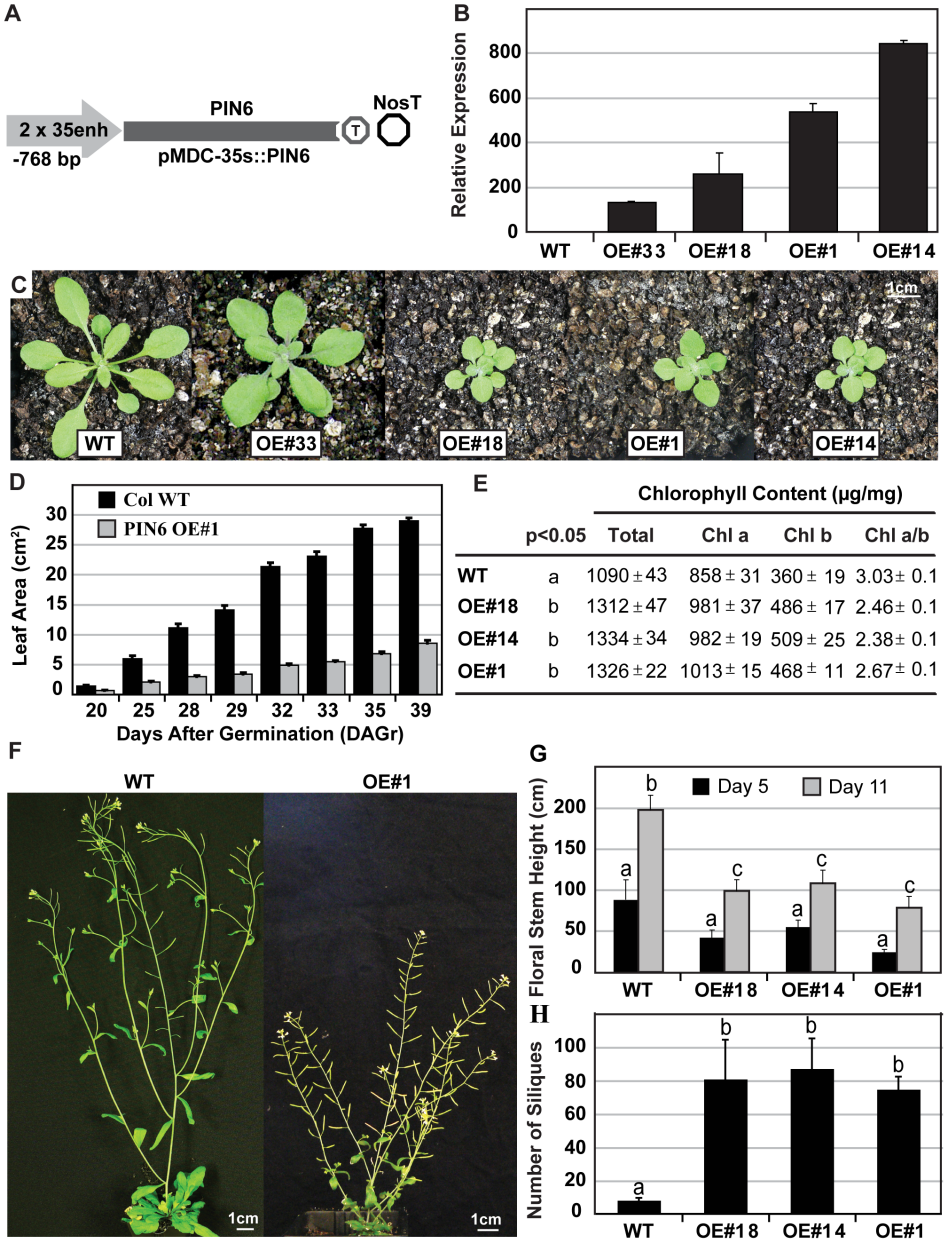
*PIN6* overexpression affects leaf pigmentation, shoot development and silique number. A) *PIN6* overexpression binary cassette (pMDC32::PIN6). B) *PIN6* mRNA abundance in leaf tissues from four representative lines and normalised to wild type (WT). C) Developing rosettes (4 weeks) from wild type and PIN6-OE lines. D) Rosette leaf area was reduced throughout vegetative development. E) Chlorophyll content was enhanced in mature leaf tissues (µg/g fresh weight). Average±SE (n = 8 plants). F) Floral architecture is modified in overexpression lines. G) Floral stem length is reduced in overexpression lines (n = 2–8 plants±SE). (H) *PIN6* overexpression enhances the production of siliques (n = 8 plants±SE). These results are representative of 2–3 independent experiments. When necessary homogeneity groups according to Student’s *t* test (p<0.05) are denoted with same letters. Error bars represent standard error.

To understand whether mis- or overexpression of *PIN6* could interfere with auxin related growth processes, we created PIN6 over-expressor lines using the strong 35S promoter (Fig. 3A). Representative transgenic lines displaying increased *PIN6* transcript abundance were selected (Fig. 3B) and the three highest overexpressing lines (PIN6-OE) lines #1, #18 and/or #14 were used for subsequent analyses. Overexpression of *PIN6* resulted in a compact rosette phenotype (Fig. 3C) and reduced leaf area (Fig. 3D). The relative leaf area expansion rate of *PIN6*-OE plants was less during the first four weeks of growth when compared to wild type (Fig. S4A). PIN6-OE leaf tissues appeared dark green and accordingly the chlorophyll content was higher on a fresh weight basis than in wild type and a reduction in the chlorophyll *a*/*b* ratio was observed (Fig. 3E). PIN6-OE lines flowered 5 to 10 days later, however the number of rosette leaves at the emergence of the floral bolt was not significantly different from wild type (Fig. S4B and S4C). Floral development of PIN6-OE lines appeared stunted when compared to wild type (Fig. 3F). PIN6-OE lines showed one or two additional rosette and cauline branches (Fig. S4D and S4E). The overall floral stem height of PIN6-OE lines was considerably smaller when compared to wild type (Fig. 3G) and there was a significant increase (∼10 fold) in the number of reproductive siliques along the entire length of the primary floral stem (Fig. 3H). These observations suggest that rosette development, chlorophyll content, fecundity and shoot apical dominance are affected by *PIN6* overexpression.

### PIN6 Overexpression Affects Root Waving, Lateral Root Development and Root Hair Outgrowth

**Figure 4:**
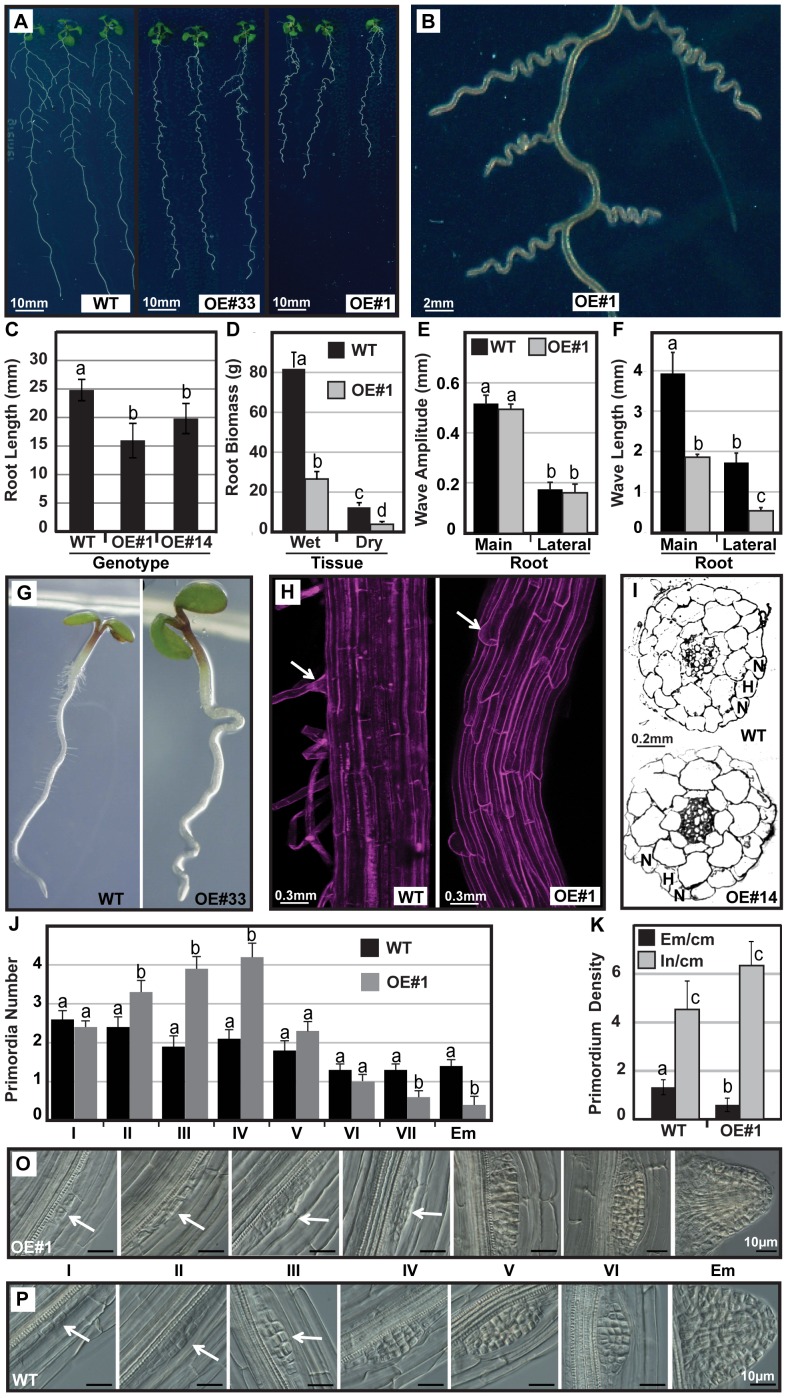
*PIN6* overexpression affects organ development and waving in roots. A). Root architecture of wild type and PIN6-OE (#33 and #1) seedlings (10 DAG). B) Close up of PIN6-OE#1 waving roots. C) PIN6-OE lines show a reduction in root elongation (10 DAG). D) Root biomass of plants growing in soil was quantified 4 weeks after germination. Average±SE (n = 12). E) and F) Wave amplitude and wave length, respectively, for primary roots. Error bars show standard error (n = 9 to 133) from a single experiment representative of three independent experiments. G) Root hair outgrowth (4 DAG) is disrupted by *PIN6* overexpression, when compared to wild type. H) Confocal micrographs show the development of trichoblast cells (PIN6-OE#1) or emergence of root hairs in WT. White arrows indicate root hair cells and PIN6-OE#1 is representative of multiple lines examined. I) Cross section of the primary root from wild type and PIN6-OE#14, respectively showing the position of a H (hair) cell in between to two N (no-hair) cells. Similar results were obtained for PIN6-OE#1. J). Development stage distribution of lateral root primordia initiated (I-VI) as well as emerged (Em) (9 DAG; n = 15) representative of multiple experiements. K) Primordium density of lateral root initiation (In) and emergence (Em) per cm of root length (8 DAG; n = 20). O) and P) Cellular patterning of root primordium at different developmental stages from initiation to emergence in PIN6 OE#1 and wild type (WT). All scale bars represent 10 µm and images are representative of multiple primordia examined. When necessary homogeneity groups according to Student’s *t* test (p<0.05) are denoted with same letters. Error bars represent standard error.

**Figure 5:**
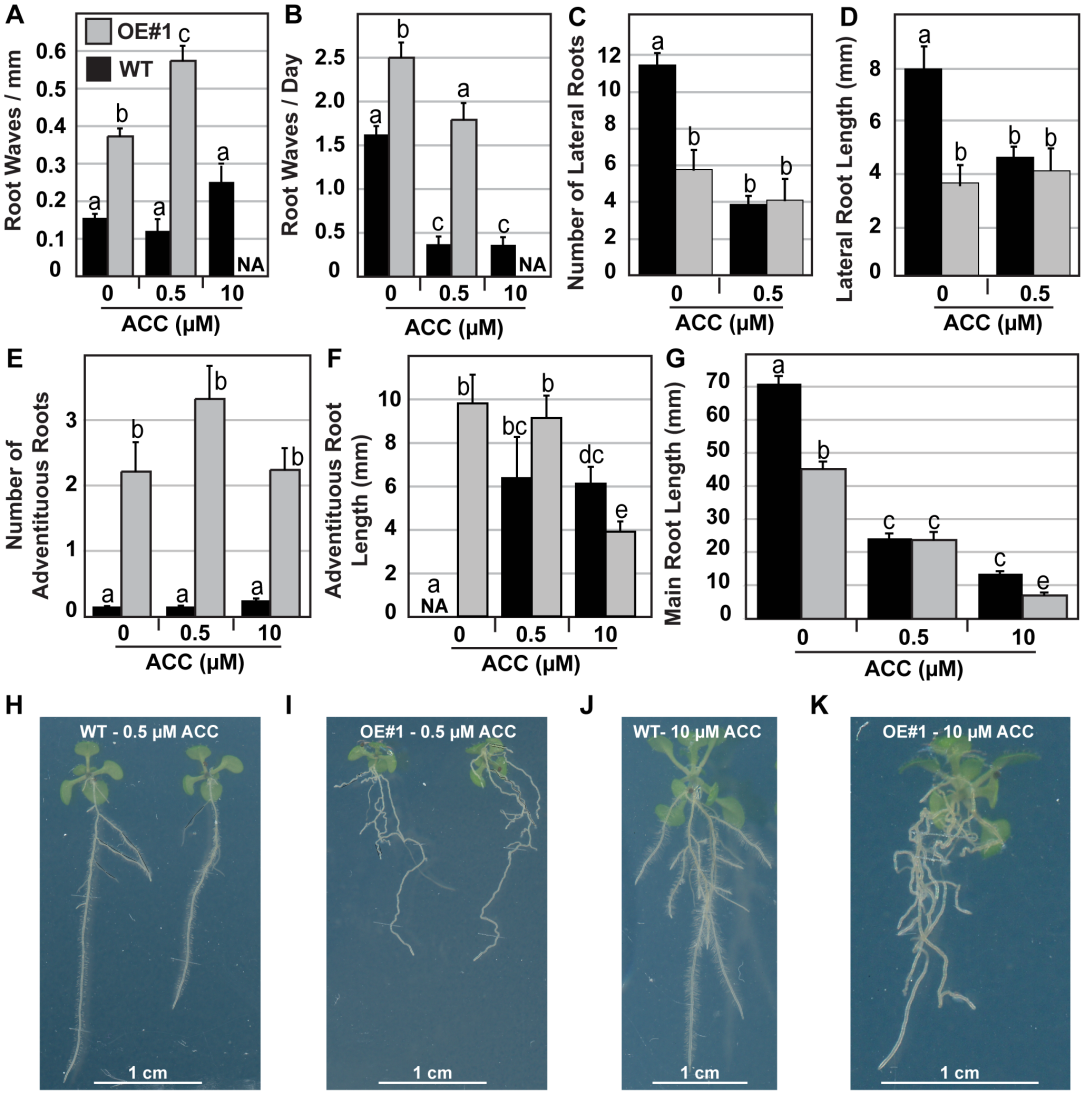
*PIN6* overexpression perturbs ethylene signalling. A) to B) Root waving was quantified in terms of number of root waves per mm (spatial waving frequency) and number of waves formed per day (temporal waving frequency), in A) and B) respectively (n = 9 to 133±SE). Misexpression of *PIN6* reduces lateral root emergence (C), perturbs root elongation (D), enhances adventitious root formation (E) and has not effect upon adventitious root length (F). Average±SE (n = 9–26). For some experiments, seedlings were germinated on media containing concentrations of the ethylene precursor, ACC. G) PIN6-OE lines show a reduction in root elongation as well as reduced sensitivity to ACC. H) to K) Responses of PIN6-OE to ACC show that the root waves became distorted, indicating altered gravitropism and sensitivity to ethylene compared to wild type. When necessary homogeneity groups according to Student’s *t* test (p<0.05) are denoted with same letters. Error bars represent standard error.

PIN6-OE interfered also with root growth and development (Fig. 4). The most obvious phenotypes were a pronounced root-waving phenotype (Fig. 4A and B) and a significant reduction in root length (Fig. 4C), which translated into much lower root biomass (Fig. 4D). All roots showed waves of similar amplitude (Fig. 4E), but instead of the regular, loose waves exhibited by wild type roots, *PIN6*-OE roots displayed much tighter waves (Fig. 4F), occurring at significantly greater frequency, in both spatial and temporal terms (Fig. 5A and B). Clearly, misexpression of *PIN6* altered the root oscillatory elongation patterns.

Another striking feature was the absence of root hair outgrowths (Fig. 4G). Confocal microscopy of the zone of cell differentiation showed that trichoblast outgrowths were absent (Fig. 4H), but a cross-section of the root showed the expected pattern of trichoblast (H cells) and atrichoblast (N cells) cells in PIN6-OE lines (Fig. 4I). Since root hair growth is proportional to internal auxin levels in the root hair cell [59], the data reveal that overexpression of *PIN6* might interfere with auxin availability in these cells.

Next we examined lateral and adventitious root development, both known to involve auxin signalling and homeostasis [34], [60]. PIN6-OE seedlings produced fewer lateral roots (Fig. 5C). As observed for the primary root, lateral roots were shorter than in wild type (Fig. 5D). Stage distribution of lateral root development (Fig. 4J) and the comparison between lateral root initiation and emergence densities (Fig. 4K), indicated a delay in lateral root emergence. Accordingly, the cellular patterning of lateral root primordia was altered by PIN6 overexpression when compared to the wild type (compare Fig. 4O with 4P). The dome shape of the lateral root primordia is missing and the regular pattern of cell divisions in developing primordia is perturbed. Eventually lateral roots emerge but the accumulation of early stage lateral root primordia and decrease in late stage primordia reveal some delay in development.

Finally, wild type seedlings occasionally showed one short adventitious root, while PIN6-OE seedlings produced 2 to 3 adventitious roots (Fig. 5E), which were well elongated (Fig. 5F). Strikingly, the root waving was especially obvious in both lateral and adventitious roots, which showed regular and spatially even waves, while in the primary root waving was more irregular and intermittent (Fig. 4B). In conclusion, misexpression of *PIN6* interferes with root elongation and waving, adventitious and lateral root development, as well as root hair outgrowth, all of which represent root growth processes dependent on the appropriate auxin levels in the respective tissues.

### PIN6 Overexpression Perturbs Ethylene Response

The phytohormone ethylene has been implicated in the control of root tropisms as well as waving [61] and has been shown to alter polar auxin transport [62]. We therefore tested whether exogenous application of 1-aminocyclopropanecarboxylic acid (ACC), the immediate precursor of ethylene, would affect the PIN6-OE elongation rate and root-waving habit (Fig. 5). Untreated PIN6-OE roots were significantly shorter than the wild type counterparts, while on ACC, their length was comparable to that of wild type roots, revealing decreased sensitivity of PIN6-OE root elongation to ethylene (Fig. 5G). ACC affected the waving of wild type roots, with a clear decrease in temporal waving frequency, but a subtle increase in the number of waves per mm of root (Fig. 5A and B), reflecting slower root elongation rates at higher concentrations of ACC (10µM). Responses of PIN6-OE lines followed a similar pattern but differed in magnitude (Fig. 5A and B); however, in contrast to wild type the root waves became quite distorted, irregular and somewhat erratic, indicating altered gravitropism and sensitivity to ethylene compared to wild type (Fig. 5H to K). ACC reduced the number and length of lateral roots found on wild type but not PIN6-OE plants (Fig. 5C and D), suggestive of a reduced sensitivity of lateral root elongation to ethylene as observed for the primary root (Fig. 5G). Adventitious root formation was not significantly affected by ACC (Fig. 5E), however in PIN6-OE plants higher concentrations of ACC (10µM) caused those roots to elongate more slowly (Fig. 5F), although this effect was much less severe than in the primary root (compare Fig. 5F and 5G). Overall these results reveal differential effects of *PIN6* overexpression on ethylene sensitivity during different developmental processes and provide evidence of a perturbed ethylene-auxin interaction in the control of the underlying cellular processes.

### PIN6 Affects Auxin Homeostasis, Accumulation and Transport

**Figure 6:**
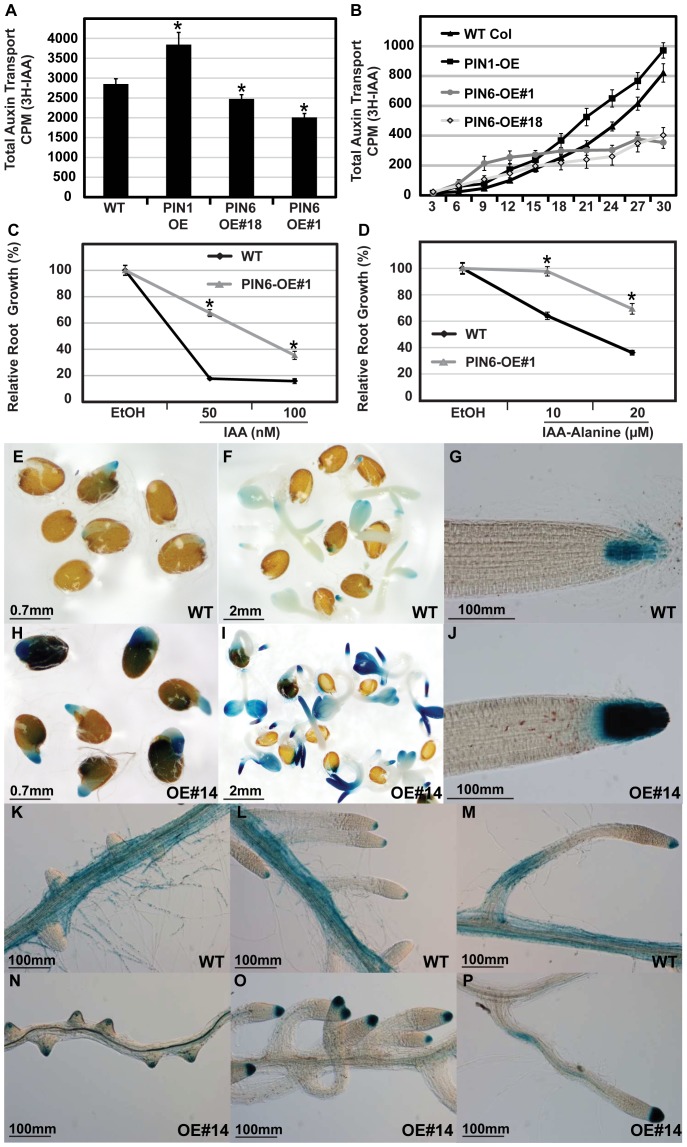
PIN6 perturbs inter-cellular auxin homeostasis in the stem, roots and cotyledons. A) Total transport of ^3^H-IAA through an inflorescence stem section. Total auxin transport was calculated by adding the radioactivity determined for each 3mm segment isolated from a 30mm stem section. Average±SE (n = 4–12 independent stem sections). B) Auxin transport profile showing movement of ^3^H-IAA through the inflorescence stem segments 3 hours after loading labelled auxin. PIN1-OE refers to a PIN1 overexpressing line. The auxin transport results presented are representative of at least three independent experiments. C) and D) Root growth assays indicate that PIN6-OE#1 is less sensitive to IAA and IAA-alanine when compared to WT (Student’s *t* test *p<0.05, n = 30 plants, Error bars represent standard deviation). E) to P) *PIN6* overexpression enhances auxin accumulation in specific tissues. Histochemical GUS staining of DR5::GUS activity in germinating seeds (E, H), 3 day old seedlings (F, I) and mature primary root tips (G, J). K) to P) GUS staining of auxin treated (200 nM NAA) root tissues from WT (Fig. 5K to 5M) and PIN6-OE#14 (Fig. 5N to 5P) harboring the DR5::GUS reporter. Where appropriate, asterisks indicate a statistically significant difference (Student’s *t* test p<0.05).

As *PIN6* overexpression interfered with auxin regulated growth responses, we tested its effects on auxin transport, homeostasis and accumulation. An isolated inflorescence stem internode assay was used to assess the in vivo function of PIN6 as a polar auxin transporter [44]. The total amount of auxin (^3^H labelled IAA) transported through the floral stem segments (similar thickness between the first and second cauline nodes) was significantly reduced in PIN6-OE lines when compared to wild type (Fig. 6A). In contrast, the PIN1 overexpression line [9] showed a significant increase in total auxin transport (Fig. 6A). In PIN6-OE plants, the basipetal wave of radioactively labelled auxin showed a slight increase in auxin transport in the stem segments closest to the ^3^H-IAA source (6–15 mm), but a significant decrease in the more distal segments (18–30 mm) (Fig. 6B). The PIN1-OE line displayed a basipetal wave similar to that of wild type (Fig. 6B). The decrease in basipetal auxin transport through the PIN6-OE stem could explain the observed defect in apical dominance in the *PIN6* overexpressing plants (Fig. 3).

A relative root growth assay showed that the PIN6-OE seedlings were less sensitive to IAA and IAA-alanine (Fig. 6C and D). High concentrations were used for the IAA-alanine conjugate because it needs to be hydrolysed (in the ER) first to IAA before it can inhibit root length [45]. An auxin inducible *DR5::GUS* reporter system [63] showed that when compared to wild type, stronger DR5 activity was observed in the PIN6-OE root tips and cotyledons during the early stages of germination (Fig. 6E, F, H and I), as well as in mature seedling root tips (Fig. 6G and J). The application of 200 nM 1-naphthaleneacetic acid (NAA) promoted strong GUS staining in the cortex and epidermis of wild type roots (Fig. 6K to M), but not in PIN6-OE roots (Fig. 6N to P), which showed stronger DR5 activity only in the tips of the emerged lateral roots (Fig. 6K to P). This shows that overexpression of *PIN6* interferes with the generation of DR5 auxin activity maxima in the roots. A stronger auxin response maximum in PIN6 overexpressing roots and root growth resistance to IAA and IAA-alanine suggest that PIN6 overexpression might interfere with accumulation of auxin in elongating epidermal and cortical cells.

In conclusion, analysis of PIN6 overexpression revealed a role of PIN6 in regulating auxin transport and auxin homeostasis.

## Discussion

### PIN6 Promoter Activity is Regulated in a Tissue-specific and Auxin Dependent Manner

In general, *PIN6* expression was relatively low throughout plant development and only detected in particular tissues during specific stages of growth. For instance, in mature embryos, *PIN6* was expressed in the root tip and root vasculature, whereas *PIN6* expression in these tissues was relatively low in seedlings. Here, *PIN6* expression was associated with the margins of the lateral root primordia as shown previously [20] and accumulated asymmetrically during root growth. *PIN6* expression levels were generally strongest in meristematic tissues and at the boundaries of root and floral organs (Fig. 1). The low expression levels of *PIN6* are in accordance with the presence of the epigenetic mark H3K27m3 that was enriched surrounding the *PIN6* promoter and gene regions. H3K27m3 has been shown to reduce gene expression levels and could contribute to the low *PIN6* transcript levels (Figure S2). Auxin induced *PIN6::GUS* expression in the shoot meristem, inner root tissues of the stele (including the pericycle), endodermis and root apical meristem (Fig. 1). Consistent with these observations a number of auxin response elements were identified within the *PIN6* promoter. Therefore, although *PIN6* is relatively weakly expressed in most tissues, the *PIN6* promoter is responsive to developmental cues in a tissue-specific and auxin-dependent manner.

The most important outstanding question concerns the subcellular localization of PIN6. PIN proteins with longer central hydrophilic loop have been shown to be localized at the PM, whereas so called shorter PINs with reduced hydrophilic loop are localized to the ER [12]. PIN6 in terms of protein topology represents an intermediate type of PIN proteins with middle-sized hydrophilic loop and thus it is difficult to envision how this type of PIN protein will be localized. The transient expression of PIN6-GFP in tobacco cells suggests an intracellular, non-PM localization of PIN6 [10]. In accordance, the *PIN6* gain-of-function analysis showing effects on auxin homeostasis and largely distinct effects on auxin transport as compared to *PIN1* gain-of-function (Fig. 6A and B) support a scenario that PIN6 is playing a role distinct from PM-localized PIN proteins. Nonetheless, the definitive answer to the *in vivo* subcellular localization of PIN6 awaits future characterisation.

### The Loss of PIN6 Function Affects Lateral Root Development

None of the identified *pin6* mutants in this study affected auxin regulated root growth. Accordingly, *PIN6* mRNA levels were not or only slightly reduced in the *pin6-4* and *pin6-5* mutants. The *pin6-6* T-DNA insertion is predicted to remove at least 22 amino acids from the 3′ end of the PIN6 protein. Although full *PIN6* transcripts, (flanking the T-DNA insertion) were not detected, the observed transcripts in a region upstream to the T-DNA insertion together with a lack of root phenotype suggest that these 22 last amino acids do not contribute significantly to the functionality of the protein. In contrast, the complete loss of PIN6 activity in *pin6-2* allele [40] did affect root growth and lateral root development. Less primordia in the early stages, together with a higher proportion of primordia in the later developmental stages, suggest a faster development of lateral roots in absence of *PIN6*. Accordingly, *PIN6* promoter activity was recorded throughout all the different stages of lateral root organogenesis. Together, the data indicate that auxin regulated root growth, in particular lateral root development requires PIN6 activity.

### PIN6 Overexpression Alters Auxin Homeostasis and Plant Development

Misexpression of *PIN6* using the CaMV 35S promoter showed that PIN6 also interferes with other growth processes that require correct distribution of auxin. In photosynthetic tissues, the overexpression of *PIN6* increased leaf chlorophyll content, promoted a compact rosette, reduced floral stem height and enhanced the emergence of seed nurturing siliques (Fig. 3). In the root, *PIN6* overexpression promoted adventitious root formation and root waving, while also perturbing root elongation, lateral primordia development as well as inhibiting the emergence of trichoblast hair cells (Fig. 4). Auxin has been shown to promote adventitious root formation and while the RNA interference of *OsPIN1* significantly inhibited adventitious root development in rice [60], [64], the overexpression of *AtPIN6* promoted the formation of adventitious roots (Fig. 5). The root-wave phenotype has been well characterised in the main Arabidopsis root [25], [61] being linked with distortion of polar auxin transport. Waving of lateral roots has been linked to light/dark cycles [65], while waving of adventitious roots has not yet been reported. The very pronounced waving of lateral and adventitious roots compared to the main root, and accumulation of auxin in the root wave crests, indicate altered auxin gradients and homeostasis interfering with the endogenous root tropisms. As noted above *pin2* mutations reduced root waving [27], while to date the overexpression of PIN proteins has not enhanced root waving, revealing this is likely to be specific to PIN6.

Overexpression of *PIN6* in lateral root primordia also indicated that misexpression of *PIN6* interferes with primordia development. In *PIN6* overexpressing lines, more primordia in the early and less primordia in the later stages of development were observed. This was consistent with a perturbation in the cellular patterning of root primordia caused by *PIN6* overexpression (Fig. 4) and contrasts nicely with the *pin6* loss-of-function phenotype, where a faster development was observed (Fig. 2). The overexpression of *PIN6* affected the auxin response maximum in developing primordia, indicating that altered auxin homeostasis induced by the overexpression of *PIN6* can interfere with lateral root development.

The tubular outgrowth of a root epidermal cell that produces a root hair can be divided into three stages, 1) fate determination to become a hair (H) or nonhair cell (N), 2) hair initiation, and 3) hair elongation [66]. Auxin is a potent stimulator of trichoblast initiation and elongation but appears to have much less effect upon cell fate–determining process [66], [67]. The overexpression of PIN6 perturbed the outgrowth of the root hair cell, however did not affect fate determination as sections from the PIN6-OE primary root showed potential H and N cells, typical of wild type roots (Fig. 4). The perturbation of polar auxin efflux through the overexpression of other long- and short-looped PINs as well as PID proteins [59], [68] was also shown to affect root hair development. It seems likely that *PIN6* overexpression reduces the level of auxin in the trichoblast cell. This would be consistent with the hypothesis that auxin efflux inhibits, and auxin influx enhances, the outgrowth of the root hair cell [69].

Evidence that *PIN6* misexpression modulates auxin homeostasis during growth and development was also provided by the altered DR5 auxin response activity in roots overexpressing *PIN6* and the failure of auxin to induce *DR5::GUS* activity in the outer cell layers of the root, leading to different GUS expression patterns (Fig. 1 and 6). Consistent with these observations our root growth assays confirmed a lower sensitivity to auxin conjugates when compared to wild type (Fig. 6). Given that PIN6 might localize to the ER, where enzymes involved in IAA metabolism are compartmentalized [70] it could be involved in regulating cellular auxin homoeostasis, presumably by compartmentalizing auxin between the ER and the cytosol and thereby fine-tune the cellular free IAA levels.

PIN6 was shown to function as an auxin (IAA) transporter in tobacco Bright Yellow-2 cell lines [9]. Here we show that overexpression of *PIN6* reduces total auxin transport through the Arabidopsis inflorescence stem (Fig. 6). The slight increase in the basipetal wave of radioactively labelled auxin nearest the ^3^H-IAA source relative to the significant decrease in the more distal segments, perhaps reflects a role for PIN6 as a regulator of inter-cellular movement of auxin efflux *in vivo*. For example, PIN6 could transport auxin in the opposite direction or in a non-polar manner, alternatively it might sequester auxin intracellularly by transporting auxin into ER thus limiting its availability for intercellular transport. Future experiments should address how *PIN6* overexpression affects auxin transport activity.

### PIN6 Misexpression Affects Ethylene Responses

Auxin and ethylene synergistically interact to coordinate root elongation with both hormones inhibiting the elongation of cells leaving the RAM [71], [72], [73], [74] (Fig. 5). Ethylene induced inhibition of root growth was reduced by *PIN6* misexpression as the root length in the PIN6-OE lines was less sensitive to ACC than in wild type, for both primary and lateral roots (Fig. 5). Interestingly, the reduction in root length appeared to be caused by early inhibitory effects of PIN6 in the first 10 days of growth, as the relative elongation rate thereafter was similar between overexpression and wild type lines (data not shown). Ethylene was shown to reduce temporal wave frequency significantly on nutrient-supplemented agar [61]; so did ACC here in both wild type and *PIN6* overexpressing lines (Fig. 5). The misexpression of *PIN6* however caused waves to become distorted, asymmetric and irregular, with the trajectory of root elongation showing strong deviations from the vertical. Therefore, the overexpression of *PIN6* reduces the sensitivity of the root to ethylene.

Ethylene regulates root growth by stimulating auxin biosynthesis and basipetal auxin transport toward the elongation zone, where it activates a local auxin response leading to inhibition of cell elongation [73], [74]. In mutants affected in basipetal auxin transport, ethylene cannot activate the auxin response or inhibit root elongation [74]. Given that *PIN6* overexpression lines have some resistance to ethylene (Fig. 5) it is possible that the misexpression of *PIN6* interferes with basipetal auxin transport. Consistently, a strong auxin response maximum at the root tip in *PIN6* overexpressors (Fig. 1 and 6), resembles auxin accumulation pattern in *pin2*/*eir1-1* mutants reported to have defects in basipetal auxin flow in the root [17]. Collectively, these data demonstrate that the misexpression of *PIN6* can perturb auxin transport dependent ethylene regulation of root growth.

## Conclusion

The *pin6* loss-of-function and misexpression lines demonstrated that PIN6 in Arabidopsis plays important developmental and physiological roles. We demonstrated that *PIN6* expression can be regulated by auxin and developmental cues and that PIN6 modifies auxin transport resulting in altered auxin dependent processes, especially those required for root growth and reproductive development.

## Supporting Information

Figure S1 Regulation of PIN6::luciferase expression during development. A) Luciferase activity spectrum for multiple independent *PIN6* transgenic lines harbouring pTPIN6::FiLUC promoter:luciferase fusion were analysed for luciferase activity and are presented in the order of increasing activity along the X-axis. Select lines chosen for further analysis of PIN6::FiLUC regulation after germination are displayed. The luciferase activity of mature leaf tissues (at least 2 leaves/plant) from independent transgenic lines (T_0_ plants) were measured using the *in vivo* leaf-disk assay (at least 2 leaf discs/leaf). B) to D) Regulation of the *PIN6* promoter during seedling development. *In vivo* bioluminescence was assayed in seedlings (B; 9 DAG), plantlets (C; 20 DAG) and flowering tissues (D; 45 DAG) from transgenic lines harbouring *PIN6* (pTPIN6::FiLUC) or *CaMV 35S* (pT35enh:FiLUC) promoters. E) False colour images obtained using the Image J software: the threshold was adjusted such that black indicates no luminescence, blue reflects low levels of luminescence and white reflects higher levels of luminescence.
(PDF)

Figure S2 Characterisation of *PIN6* expression patterns. A) PIN6 anatomical mRNA expression levels in wild type Columbia-O tissues. Genevestigator was used to generate an Arabidopsis PIN6 transcript profile across a range of tissues and values >400 are considered to have medium expression levels [55]. B) and C) Quantification of *PIN6* promoter-GUS activity in mature leaf and stem tissues, respectively. Tissues were harvested 28 DAG from multiple independent lines (n = 21) and GUS activities expressed in nmoles 4MU/min/mg of soluble protein. Lines are presented in the order of increasing activity along the X-axis. Leaf error bars (B) represent±SE of three independent experiments (n = 3) measuring pooled tissues from a single plant (hemizygous) in duplicate. Primary stem tissues were pooled from a single hemizygous plant and assayed in duplicate (SE bars not shown). A strong expressing CaMV 35S::GUS line was included as a positive control and the average of the PIN6::GUS lines is displayed. Representative lines chosen for further analysis are displayed above the bars. D) *PIN* gene expression levels during plant development. GENEVESTIGATOR was used to collate published microarray data and report expression levels in germinating seeds, young seedlings, mature rosettes, floral bolts as well as young immature and older mature flowers. E) Summary of *PIN* expression, chromatin modifications and protein localisation. GENEVESTIGATOR expression levels were qualitatively scored as strong (+++), medium (++) and weak (++) and H3K27 trimethylation marks associated with repressive *PIN* gene expression were scored as absent (no) or present (yes) (http://www.mcdb.ucla.edu/Research/Jacobsen/). Subcellular targeting of the *PIN* genes to the plasma membrane (PM) or endoplasmic reticulum (ER) are shown.
(PDF)

Figure S3 Characterisation of additional *pin6* mutant alleles. A) End-point PCR was used to quantify *PIN6* expression levels in 10 day old seedling tissues from TDNA insertion and overexpression lines. Mutant *pin6–5* showed slightly reduced *PIN6* mRNA levels, while OE#1 showed increased *PIN6* transcript levels. B) to D) RT- PCR was used to measure *PIN6* mRNA abundance in *pin6-6* seedling (10 DAG) and/or flowering tissues. PCR amplicons either flanked (B) or positioned before the GK_711C09 TDNA insertion (C). D) *ACTIN* was used as a control to quantify cDNA.
(PDF)

Figure S4 PIN6 overexpression affects floral development. A) Relative rosette expansion rate of PIN6-OE#1 compared to wild type. The asterisk indicates the onset of floral bolt emergence. Relative growth rate = Log{Area (T_2_)}–Log {Area (T_1_)}/(T_2_ – T_1_). The average±SE (n = 8) are given. B) and C) Days to flowering and number of leaves at the onset of flowering, respectively. ND = not determined. D) and E) The number of rosette and cauline branches, respectively. The number of rosette branches excludes the main primary floral bolt and only cauline branches emerging from the primary floral stem were scored. The average±SE (n = 7–8) are given. Statistically significant data are indicated by a star (*t-*tests, 2 tailed, unpaired, P<0.05). Similar data were observed in multiple experiments under a range of experimental conditions.
(PDF)

Table S1 Primers used to quantify transcript abundance and construct binary vectors. The target gene, primer name, sequence and direction of primer annealing are displayed.
(PDF)
