# Supplementary material for: Role of the Arabidopsis PIN6 Auxin Transporter in Auxin Homeostasis and Auxin-Mediated Development
Source: PLoS One. 2013 Jul 29;8(7):e70069. doi: 10.1371/journal.pone.0070069 (PMC3726503; doi:10.1371/journal.pone.0070069)
Supplement: Figure S1 — Regulation of PIN6::luciferase expression during development. A) Luciferase activity spectrum for multiple independent PIN6 transgenic lines harbouring pTPIN6::FiLUC promoter:luciferase fusion were analysed for luciferase activity and are presented in the order of increasing activity along the X-axis. Select lines chosen for further analysis of PIN6::FiLUC regulation after germination are displayed. The luciferase activity of mature leaf tissues (at least 2 leaves/plant) from independent transgenic lines (T0 plants) were measured using the in vivo leaf-disk assay (at least 2 leaf discs/leaf). B) to D) Regulation of the PIN6 promoter during seedling development. In vivo bioluminescence was assayed in seedlings (B; 9 DAG), plantlets (C; 20 DAG) and flowering tissues (D; 45 DAG) from transgenic lines harbouring PIN6 (pTPIN6::FiLUC) or CaMV 35S (pT35enh:FiLUC) promoters. E) False colour images obtained using the Image J software: the threshold was adjusted such that black indicates no luminescence, blue reflects low levels of luminescence and white reflects higher levels of luminescence. (PDF) [file pone.0070069.s001.pdf]

Figure S1

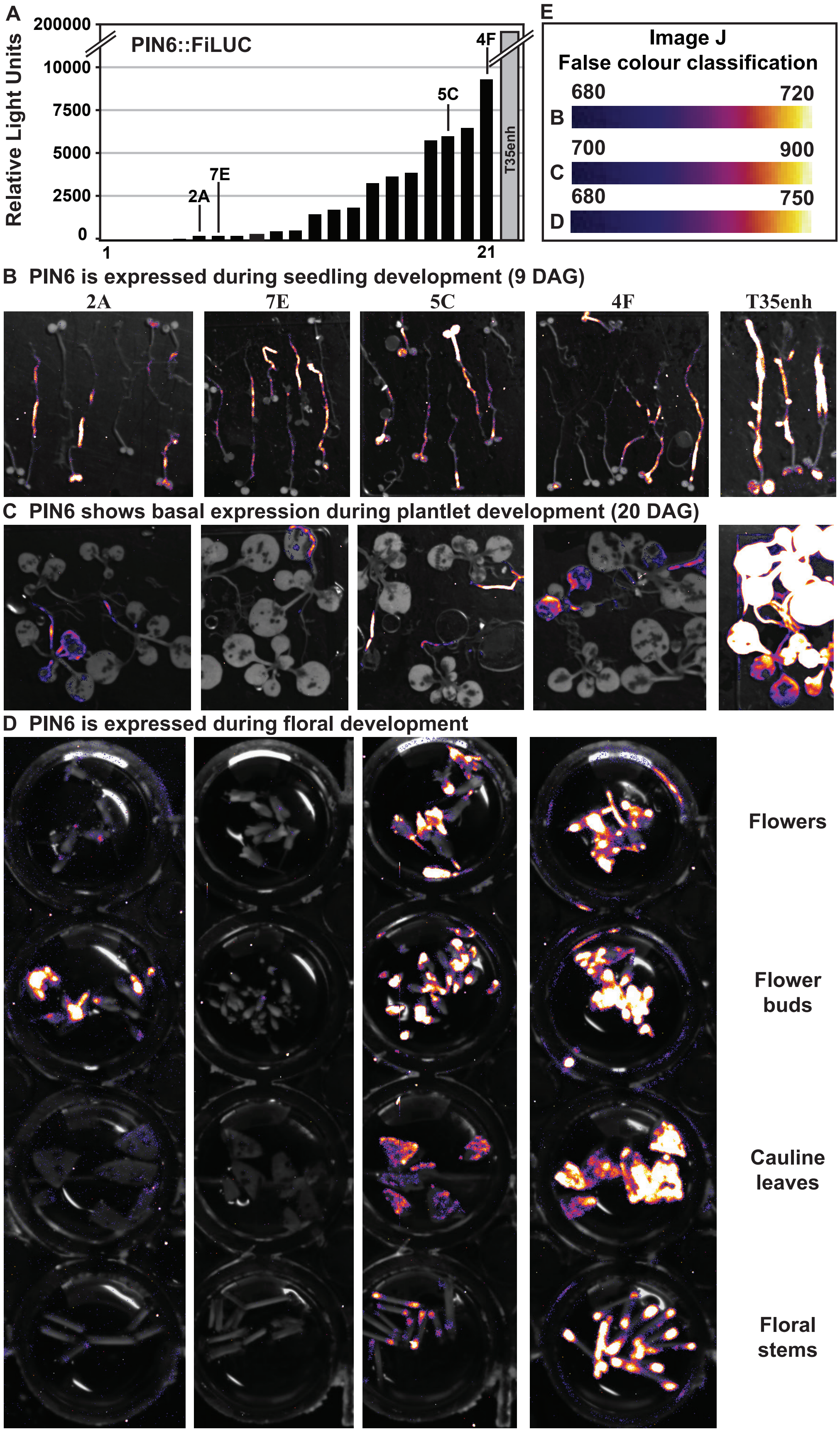

Figure S1. Regulation of PIN6::luciferase expression during development. A) Luciferase activity spectrum for multiple independent *PIN6* transgenic lines harbouring pTPIN6::FiLUC promoter:luciferase fusion were analysed for luciferase activity and are presented in the order of increasing activity along the X-axis. Select lines chosen for further analysis of PIN6::FiLUC regulation after germination are displayed. The luciferase activity of mature leaf tissues (at least 2 leaves/plant) from independent transgenic lines (T_0 plants) were measured using the *in vivo* leaf-disk assay (at least 2 leaf discs/leaf). B) to D) Regulation of the *PIN6* promoter during seedling development. *In vivo* bioluminescence was assayed in seedlings (B; 9 DAG), plantlets (C; 20 DAG) and flowering tissues (D; 45 DAG) from transgenic lines harbouring *PIN6* (pTPIN6::FiLUC) or *CaMV35s* (pT35enh:FiLUC) promoters. E) False colour images obtained using the Image J software: the threshold was adjusted such that black indicates no luminescence, blue reflects low levels of luminescence and white reflects higher levels of luminescence.
