# Supplementary figures and images for: Role of the Arabidopsis PIN6 Auxin Transporter in Auxin Homeostasis and Auxin-Mediated Development
Source: PLoS One. 2013 Jul 29;8(7):e70069. doi: 10.1371/journal.pone.0070069 (PMC3726503; doi:10.1371/journal.pone.0070069)

**Figure S2**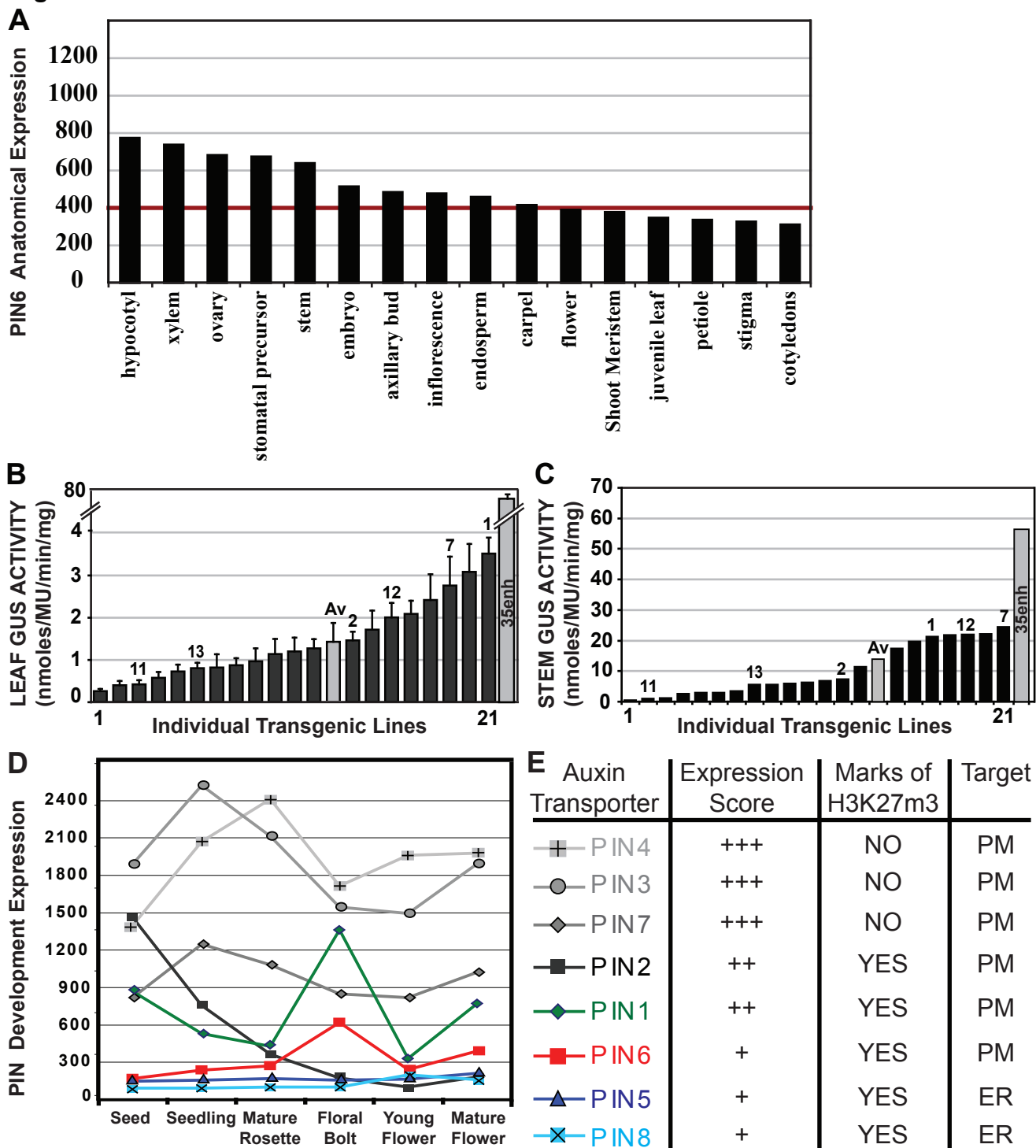

Supplement: Figure S2 — Characterisation of PIN6 expression patterns. A) PIN6 anatomical mRNA expression levels in wild type Columbia-O tissues. Genevestigator was used to generate an Arabidopsis PIN6 transcript profile across a range of tissues and values >400 are considered to have medium expression levels [55]. B) and C) Quantification of PIN6 promoter-GUS activity in mature leaf and stem tissues, respectively. Tissues were harvested 28 DAG from multiple independent lines (n = 21) and GUS activities expressed in nmoles 4MU/min/mg of soluble protein. Lines are presented in the order of increasing activity along the X-axis. Leaf error bars (B) represent±SE of three independent experiments (n = 3) measuring pooled tissues from a single plant (hemizygous) in duplicate. Primary stem tissues were pooled from a single hemizygous plant and assayed in duplicate (SE bars not shown). A strong expressing CaMV 35S::GUS line was included as a positive control and the average of the PIN6::GUS lines is displayed. Representative lines chosen for further analysis are displayed above the bars. D) PIN gene expression levels during plant development. GENEVESTIGATOR was used to collate published microarray data and report expression levels in germinating seeds, young seedlings, mature rosettes, floral bolts as well as young immature and older mature flowers. E) Summary of PIN expression, chromatin modifications and protein localisation. GENEVESTIGATOR expression levels were qualitatively scored as strong (+++), medium (++) and weak (++) and H3K27 trimethylation marks associated with repressive PIN gene expression were scored as absent (no) or present (yes) (http://www.mcdb.ucla.edu/Research/Jacobsen/). Subcellular targeting of the PIN genes to the plasma membrane (PM) or endoplasmic reticulum (ER) are shown. (PDF) [file pone.0070069.s002.pdf]
