# Supplementary material for: Role of the Arabidopsis PIN6 Auxin Transporter in Auxin Homeostasis and Auxin-Mediated Development
Source: PLoS One. 2013 Jul 29;8(7):e70069. doi: 10.1371/journal.pone.0070069 (PMC3726503; doi:10.1371/journal.pone.0070069)
Supplement: Figure S3 — Characterisation of additional pin6 mutant alleles. A) End-point PCR was used to quantify PIN6 expression levels in 10 day old seedling tissues from TDNA insertion and overexpression lines. Mutant pin6–5 showed slightly reduced PIN6 mRNA levels, while OE#1 showed increased PIN6 transcript levels. B) to D) RT- PCR was used to measure PIN6 mRNA abundance in pin6-6 seedling (10 DAG) and/or flowering tissues. PCR amplicons either flanked (B) or positioned before the GK_711C09 TDNA insertion (C). D) ACTIN was used as a control to quantify cDNA. (PDF) [file pone.0070069.s003.pdf]

Figure S3

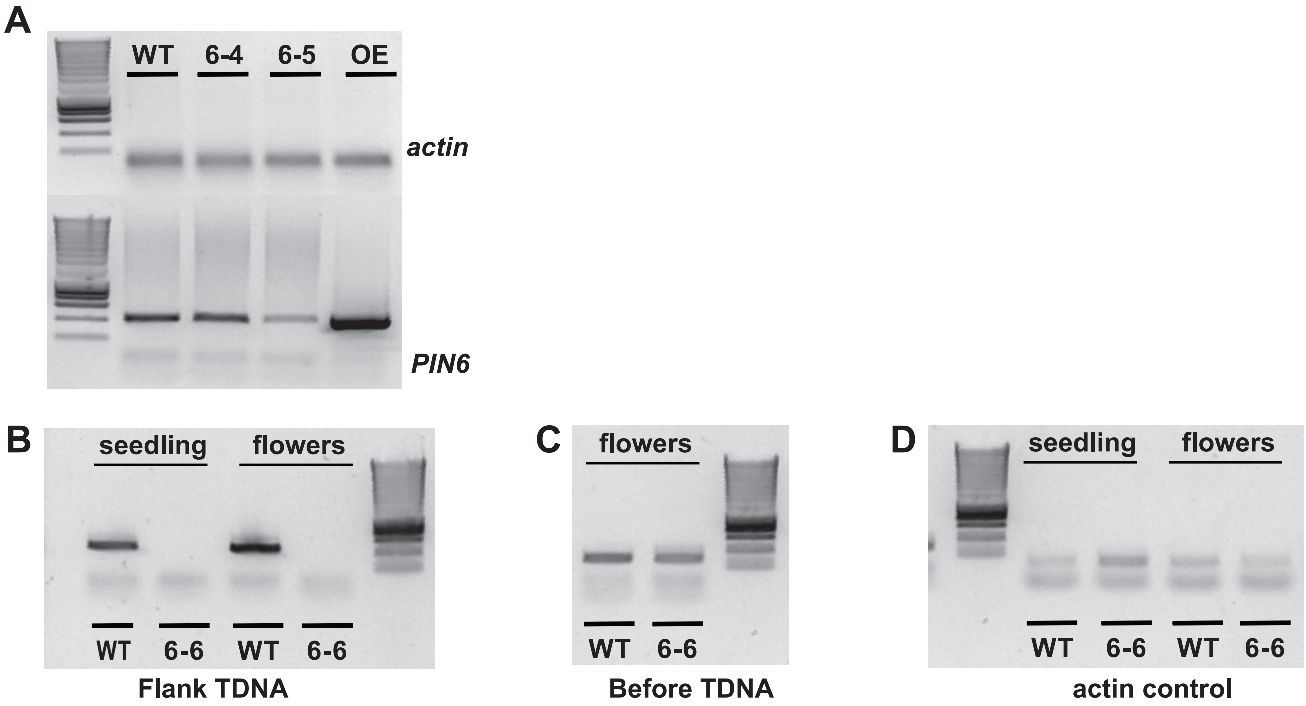

Figure S3. Characterisation of additional *pin6* mutant alleles. A) End-point PCR was used to quantify *PIN6* expression levels in 10 day old seedling tissues from TDNA insertion and overexpression lines. *pin6-5* showed slightly reduced *PIN6* mRNA levels, while OE#1 showed increased *PIN6* transcript levels. B) to D) RT- PCR was used to measure *PIN6* mRNA abundance in *pin6-6* seedling (10 DAG) and/or flowering tissues. PCR amplicons either flanked (B) or positioned before the GK_711C09 TDNA insertion (C). D) ACTIN was used as a control to quantify cDNA.
