# Supplementary material for: Role of the Arabidopsis PIN6 Auxin Transporter in Auxin Homeostasis and Auxin-Mediated Development
Source: PLoS One. 2013 Jul 29;8(7):e70069. doi: 10.1371/journal.pone.0070069 (PMC3726503; doi:10.1371/journal.pone.0070069)
Supplement: Figure S4 — PIN6 overexpression affects floral development. A) Relative rosette expansion rate of PIN6-OE#1 compared to wild type. The asterisk indicates the onset of floral bolt emergence. Relative growth rate = Log{Area (T2)}–Log {Area (T1)}/(T2 – T1). The average±SE (n = 8) are given. B) and C) Days to flowering and number of leaves at the onset of flowering, respectively. ND = not determined. D) and E) The number of rosette and cauline branches, respectively. The number of rosette branches excludes the main primary floral bolt and only cauline branches emerging from the primary floral stem were scored. The average±SE (n = 7–8) are given. Statistically significant data are indicated by a star (t-tests, 2 tailed, unpaired, P<0.05). Similar data were observed in multiple experiments under a range of experimental conditions. (PDF) [file pone.0070069.s004.pdf]

Figure S4

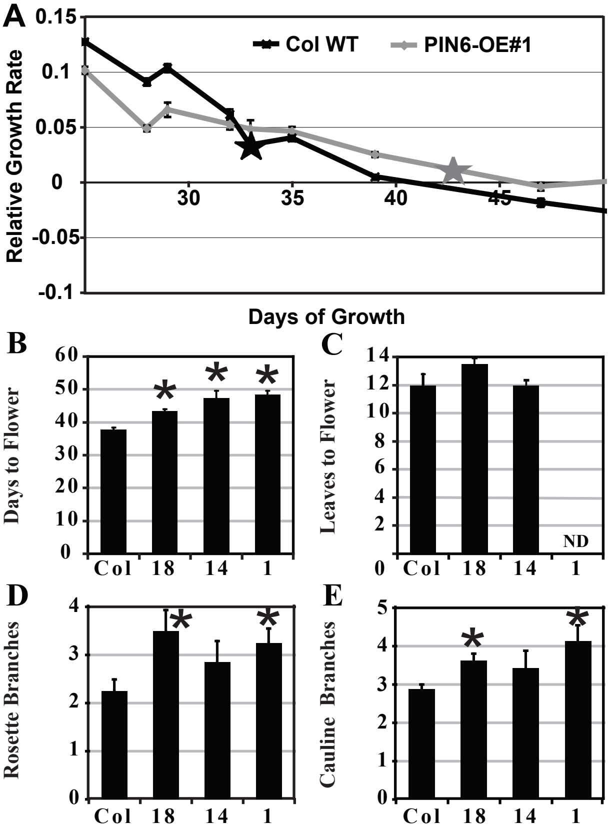

Figure S4. PIN6 overexpression affects floral development. A) Relative rosette expansion rate of PIN6-OE#1 compared to wild type. The asterisk indicates the onset of floral bolt emergence. Relative growth rate = Log{Area (T_2)} – Log {Area (T_1)} / $(T_2 - T_1)$. The average ± SE (n=8) are given. B) and C) Days to flowering and number of leaves at the onset of flowering, respectively. ND = not determined. D) and E) The number of rosette and cauline branches, respectively. The number of rosette branches excludes the main primary floral bolt and only cauline branches emerging from the primary floral stem were scored. The average ± SE (n=7-8) are given. Statistically significant data are indicated by a star (*t*-tests, 2 tailed, unpaired, $P<0.05$). Similar data were observed in multiple experiments under a range of experimental conditions.
