# Supplementary material for: Role of the Arabidopsis PIN6 Auxin Transporter in Auxin Homeostasis and Auxin-Mediated Development
Source: PLoS One. 2013 Jul 29;8(7):e70069. doi: 10.1371/journal.pone.0070069 (PMC3726503; doi:10.1371/journal.pone.0070069)
Supplement: Table S1 — Primers used to quantify transcript abundance and construct binary vectors. The target gene, primer name, sequence and direction of primer annealing are displayed. (PDF) [file pone.0070069.s005.pdf]

Table S1. Primers used to quantify transcript abundance and construct binary vectors. The target gene, primer name, sequence and direction of primer annealing are displayed.

Target	Primer Name	Sequence (5'-->3')	Direction
**House Keeping Genes**			
Cyclophilin	Cyclo	TCTTCCTCTTCGGAGCCATA<br>AAGCTGGGAATGATTCGATG	Sense<br>Antisense
Actin	Actin2	TTGACTACGAGCAGGAGATGG<br>ACAAACGAGGGCTGGAACAAG	Sense<br>Antisense
**Gene Specific Primers for qRT-PCR**			
PIN6	PIN6-CC1F<br>PIN6-CC1R	TGGGCCGTTTTCTTCAAAGC<br>GATTGATCCGGCTGCTTGAC	Sense<br>Antisense
PIN6<br>(*pin6-4*; *pin6-5*)	GB_430B01_RT1_Fw (A-f)<br>GB_430B01_RT1_Rv (A-r)	TACGCCGGTTTGGATGAAAT<br>GCATCTGAAATGATCTTTATCGAGA	Sense<br>Antisense
PIN6<br>(*pin6-6*; Prior TDNA)	GB_430B01_RT2_Fw (C-f)<br>GB_430B01_RT2_Rv (C-r)	AAGGAGATTAGCTTCAGAGA<br>CTGAAATGATCTTTATCGAG	Sense<br>Antisense
PIN6<br>(*pin6-6*; Flanking TDNA)	Pin6-RT2-Fw (B-f)<br>Pin6-RT2-Rev (B-r)	GAAAAAGGCAACAATGGGGA<br>CACCTAACCATTTACCAGTAATTTG	Sense<br>Antisense
**Overexpression Constructs**			
pMDC35enh::PIN6	PIN6 Gene-F<br>PIN6 Term-R	attB1-AAAGATGATAACGGGAAACGAA<br>attB2-TCTCACAAGCTCCTCAAGAACA	Sense<br>Antisense
Gateway Homologous recombination	attB1<br>attB2	ACAAGTTTGTACAAAAAAGCAGGCT<br>ACCACTTTGTACAAGAAAGCTGGGT	Sense<br>Antisense
**Promoter-Reporter Gene Binary Vectors**			
pTPIN6::FiLUC	PIN6Prom-cc2F<br>Pin6Prom-cc1R	GCTCTAGAGTTTGCTGCAACTGTATGC<br>CATGCCATGGCTTTTTCTTTGCCTCTTCTTC	Sense<br>Antisense
pMDCPIN6::GUS	PIN6Prom-F<br>Pin6Prom-R	attB1- GCAAAGTTTTTATATATGCTCGGAT<br>attB2- CTTTTTCTTTGCCTCTTCTTCTTCT	Sense<br>Antisense
